# The effects of hepatitis C virus core protein on the expression of miR-122 in vitro

**DOI:** 10.1186/1743-422X-10-98

**Published:** 2013-03-27

**Authors:** Sujuan Li, Xiaokang Xing, Qiao Yang, Hangdi Xu, Jiliang He, Zhi Chen, Haihong Zhu

**Affiliations:** 1 State Key Laboratory for Diagnosis and Treatment of Infectious Diseases, Institute of Infectious Diseases, the First Affiliated Hospital, School of Medicine, Zhejiang University, Hangzhou, 310003, China; 2 Institute of Environmental Medicine, Medical College, Zhejiang University, Hangzhou, 310058, China

## Abstract

**Background:**

Hepatitis C virus (HCV) is one of the major pathogens of liver diseases. Some studies have previously reported that miR-122 can stimulate replication or translation of HCV. However, the effects of HCV infection on miR-122 expression are not clear. The aim of this study was to investigate the effects of HCV core protein on the expression of miR-122 in a cell culture model.

**Results:**

The miR-122 levels in Huh7.5.1 cells infected with HCV for different days or different HCV abundance were measured by real-time PCR. Significant decrease of miR-122 expression was found at late stage of infection and in the high-abundance group. Huh7.5.1 cells transfected with plasmid pEGFP-core or pEGFP were used to detect the effects of HCV core protein on miR-122 expression, the results showed that core protein could down-regulate the miR-122 expression level in a time- and dose- dependent manner, and reduced the susceptibility of Huh7.5.1 cell to HCV.

**Conclusions:**

Down-regulating miR-122 expression by HCV core protein may give a new insight into the interaction between HCV and miR-122 and chronic HCV infection.

## Background

Hepatitis C virus (HCV) is one of the major causes of viral hepatitis and hepatocellular carcinoma, which infects an estimated 170 million people throughout the world [1,2]. Currently, pegylated interferon-α (IFN-α) combined with ribavirin is the standard of care for chronic HCV infection. The therapy has a sustained virological response rate of 40% ~ 50% in patients with genotype 1 HCV infection [3-5], which accounts for the majority of the hepatitis C patient population in the developed countries. A range of non-structural protease inhibitors are developed to act as antiviral agents against HCV, such as boceprevir [6,7] and telaprevir [8,9]. However, genetic heterogeneity between HCV genotypes influences the structure of protease and limits the effectiveness of the antivirals [10]. Most people infected with HCV develop chronic liver disease [11]. The success of HCV in persisting is linked to its overall ability to evade antiviral defences. Several HCV structural and nonstructural proteins, including Core [12,13], E2 [14], NS3/4A [15,16] and NS5A [17] proteins, have been shown to inhibit the innate immune response.

miRNAs are post-transcriptional regulators that bind to complementary sequences on target messenger RNA transcripts (mRNAs), usually resulting in translational repression or target degradation and gene silencing. miR-122, a liver-specific miRNA expressed at high levels in hepatocytes, constitutes 70% of the total miRNA in the liver [18]. miR-122 enhanced the replication of HCV through a direct interaction with the 5’noncoding region (NCR) of the HCV genome [19]. Henke et al. showed that miR-122 stimulated translation of HCV by enhancing the association of ribosomes with the viral RNA [20]. Furthermore, it was proved that up-regulation of HMOX1, which was the target gene of miR-122, decreased HCV RNA [21]. However, the effects of HCV on the expression of miR-122 haven’t been fully explored yet.

In the current study, a cell culture model based on Jc1 clone and Huh7.5.1 cell was used to investigate the effects of HCV on the expression of miR-122. Data showed that HCV decreased the expression of miR-122 in a time- and dose- dependent manner. Further results revealed that the HCV core protein performed this function. These results give a novel insight into the interaction between HCV and miR-122.

## Results

### HCV decreased the expression of miR-122 in a time- and dose- dependent manner

**Figure 1:**
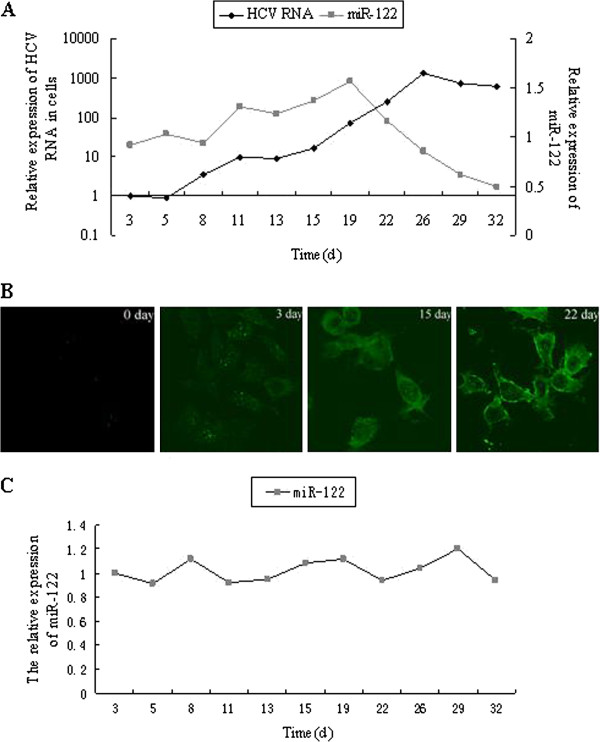
**Replication of HCV and expression of miR-122 in Huh7.5.1 cells after infection with HCV.** (**A**) Huh7.5.1 cells infected with 2 × 10^6^ IU/ml HCV particles were harvested at the indicated time points post-infection. Intracellular HCV RNA and miR-122 were analyzed by real-time PCR, using 3 days post-infection as control group. (**B**) Intracellular expression of HCV core protein was assessed by indirect immunofluorescent staining. (**C**) Uninfected huh7.5.1 cells were cultured for 32 days as parallel control group. Intracellular miR-122 was analyzed by real-time PCR, using 3 days as control group.

Huh7.5.1 cells infected with HCV Jc1 virus were used in the following studies to determine whether HCV replication would regulate the expression of miR-122. Cells were harvested at different time points, and the relative expressions of HCV RNA and miR-122 were determined by real-time PCR. Intracellular HCV RNA increased rapidly and reached the highest level at 26 days post-infection (Figure 1A), which was further confirmed by immunofluorescence staining (Figure 1B). These results suggested that HCV replicated effectively in infected Huh7.5.1 cells. miR-122 expression increased to the maximum level of 157% (vs day 3) at day 19 post-infection (Figure 1A), then decreased quickly and reached the minimum level of 50% (vs day 3) at day 32 post-infection (Figure 1A). At day 26, day 29 and day 32 post-infection, corresponding to the high levels of HCV RNA, intracellular levels of miR-122, however, decreased to 86%, 62% and 50%, respectively (vs day 3) (Figure 1A). The experiment was repeated three times, during which our observation and data were quite consistent with the Figure 1A. In contrast, there were no significant changes in expressions of miR-122 in uninfected group throughout the experiment (Figure 1C).

**Figure 2:**
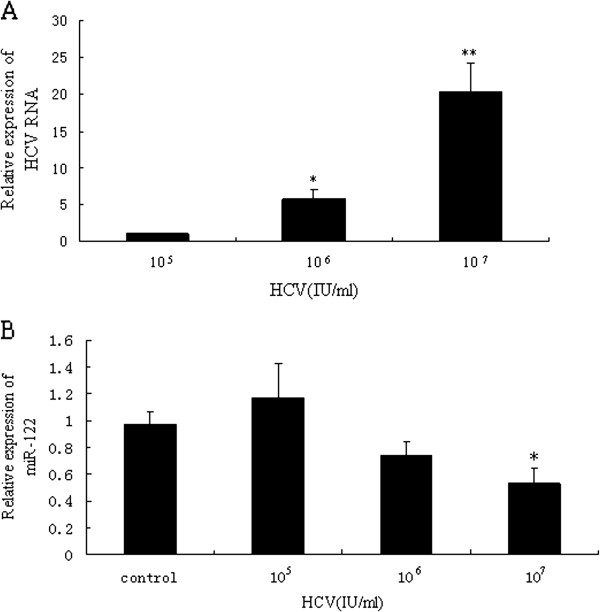
**Expressions of HCV RNA and miR-122 in Huh7.5.1 cells after infection with different levels of HCV.** (**A**) Intracellular expressions of HCV RNA in Huh7.5.1 cells infected with different levels of HCV were assayed, using 10^5^ IU/ml group as control group. (**B**) Intracellular expressions of miR-122 in Huh7.5.1 cells infected with different levels of HCV were assayed, using mock group as control group. The data were presented as means ± SD of three independent experiments. ^*^P < 0.05, ^**^P < 0.01 vs. control group.

To determine whether miR-122 expression was related with the amount of HCV, we infected Huh7.5.1 cells with different amounts of HCV for 10 days. The expression of HCV RNA in each group (10^5^ IU/ml group, 10^6^ IU/ml group and 10^7^ IU/ml group) increased to different levels (*P* < 0.05 or *P* < 0.01) (Figure 2A). We then measured the expression of miR-122 in each group. There was a ~47% decrease of miR-122 expression with statistical significance in the 10^7^ IU/ml group (10^7^ IU/ml 0.53 ± 0.12 vs control 0.97 ± 0.09 *P* < 0.05) (Figure 2B). In contrast, no significant differences were observed in the 10^5^ IU/ml group and 10^6^ IU/ml group (Figure 2B).

### HCV core protein down-regulated miR-122 expression

**Figure 3:**
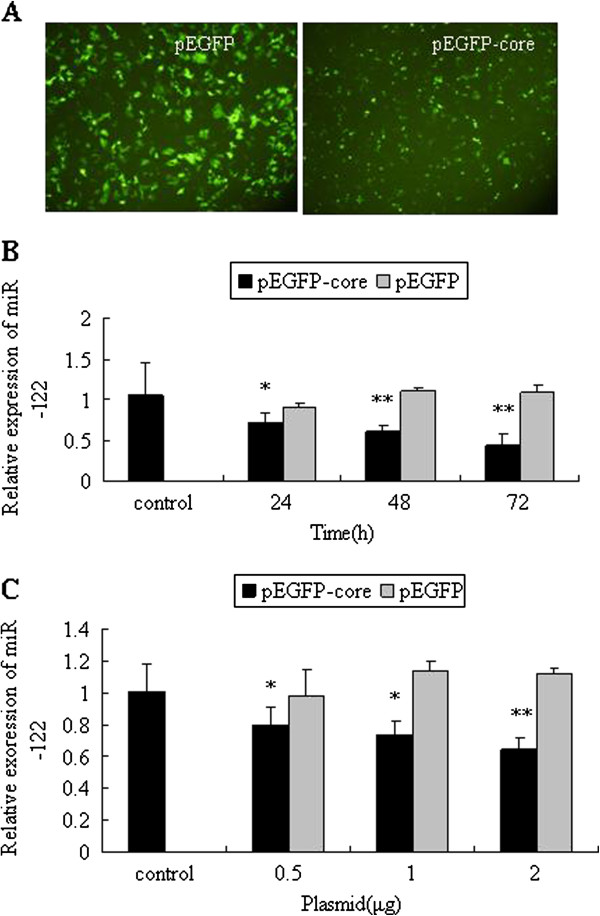
**HCV core protein suppressed the expression of miR-122.** (**A**) The intensity of EGFP in Huh7.5.1 cells transfected with plasmid pEGFP-core or pEGFP was observed by fluorescence microscopy. (**B**) Huh7.5.1 cells transfected with 1.5 μg of plasmid pEGFP-core or pEGFP were harvested at 24 h, 48 h and 72 h posttransfection. The relative expression of miR-122 was detected, using mock group as control group. (**C**) miR-122 expressions in Huh7.5.1 cells transfected with 0.5 μg, 1 μg and 2 μg of the plasmid pEGFP-core or pEGFP were detected at 48 h posttransfection, using mock group as control group. The data were presented as means ± SD of three independent experiments. ^*^P < 0.05, ^**^P < 0.01 vs. control group.

To evaluate whether HCV core protein affected the expression of miR-122, we transfected Huh7.5.1 cells with plasmid pEGFP-core expressing GFP-tagged HCV core protein or plasmid pEGFP (Figure 3A). miR-122 expression was detected in the Huh7.5.1 cells at 24 h, 48 h and 72 h post-transfection. HCV core protein suppressed miR-122 expression in a time-dependent manner (24 h 0.71 ± 0.114, 48 h 0.60 ± 008, 72 h 0.45 ± 0.121, vs control 1.06 ± 0.406, *P* < 0.05 or *P* < 0.01) (Figure 3B). The suppression effect of HCV core protein was also dose-dependent since the level of miR-122 was proportionally reduced when increasing amounts of HCV core protein were transfected into cells (0.5 μg 0.80 ± 0.112, 1 μg 0.74 ± 0.086, 2 μg 0.64 ± 0.082, vs control 1.01 ± 0.179, *P* < 0.05 or *P* < 0.01) (Figure 3C). In contrast, GFP protein had no significant effect on miR-122 expression (Figure 3B) (Figure 3C).

### HCV core protein down-regulated susceptibility of Huh7.5.1 cells to HCV

**Figure 4:**
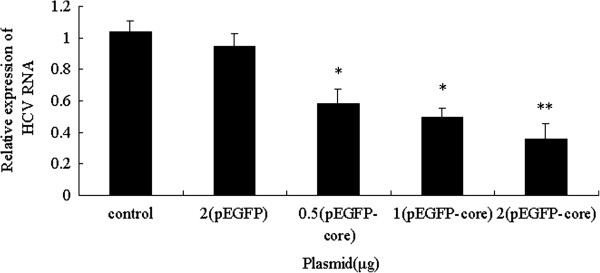
**The susceptibility of Huh7.5.1 cells to HCV after transfection with plasmid pEGFP-core or pEGFP.** Huh7.5.1 cells were transfected with 0.5 μg, 1 μg and 2 μg of the plasmid pEGFP-core or pEGFP for 48 h, and then infected with 1 **×** 10^7^ IU/ml HCV particles for 72 h. Relative level of HCV RNA was quantified by real-time PCR, using 72 h post-infection as control group. The data were presented as means ± SD of three independent experiments. ^*^P < 0.05, ^**^P < 0.01 vs. pEGFP group. *P >* 0.05 pEGFP group vs. control group.

Subsequently, Huh7.5.1 cells transfected with different doses of plasmid pEGFP-core or pEGFP were infected with HCV to investigate whether core protein influenced the susceptibility of Huh7.5.1 cells to HCV. Actually, HCV RNA levels in groups transfected with plasmid pEGFP-core decreased when compared to pEGFP group (0.5 μg 0.58 ± 0.088, 1 μg 0.49 ± 0.058, 2 μg 0.36 ± 0.098 vs control 0.94 ± 0.08, *P* < 0.05 or *P* < 0.01) (Figure 4). In contrast, there wasn’t an obvious change of HCV RNA expression in pEGFP group when compared to control group (Figure 4).

## Discussion

It is generally believed that miR-122, specifically expressed and highly abundant in the liver, facilitates replication of HCV. That is the reason why HCV RNA can only replicate in Huh7 cells, but not in HepG2 cells, which do not express endogenous miR-122 [19].

In recent years, many studies have focused on the relationship between HCV and miR-122. There is, however, only limited information about the role of HCV in miR-122 expression. In our study, the intracellular levels of HCV RNA and miR-122 were measured at different time points after infecting with HCV. The results indicated that the level of HCV RNA increased rapidly during the infection, and there was a positive correlation between miR-122 level and HCV RNA level at the early stage of infection to some extent. However, in previous reports, no positive correlation was observed between miR-122 level and HCV [22,23]. It may be due to not strong correlation between miR-122 level and HCV RNA level or the different cell line. It was interesting that at day 19 after infection with HCV, there was an inverse correlation between miR-122 expression and HCV RNA expression, and miR-122 expression level was obviously reduced at the late stage of HCV infection. This inverse correlation between HCV and miR-122 was consistent with a previous in vitro study, which reported that miR-122 levels were significantly reduced on HCVcc infection [24]. It was also found in our study that miR-122 expression level decreased in high-abundance HCV group. In a vivo experiment, a similar result was observed that the HCV subjects with higher viral load had lower miR-122 levels in liver than HCV subjects with lower viral load [25]. Another similar result was published that hepatic miR-122 expression in serum HCV RNA-positive patients was significantly lower than that in serum HCV RNA-negative patients [26]. Thus, it seemed that, with the accumulation of HCV, the inhibitory effect of HCV on miR-122 might be observed. Taken together, these results might support the notion that HCV monitors its replication by down-regulating the miR-122 level. Actually, a recently proposed hypothesis may provide a similar mechanistic explanation that, in order to survive, HCV need a steady viral replication rate [27-30]. By keeping abundance relatively low, the virus evades the immune response of the host and establishes persistent chronic infection.

The subsequent results demonstrated that it was HCV core protein that suppressed miR-122 expression both in a time- and dose- dependent manner. And overexpression of HCV core protein did reduce the susceptibility of Huh7.5.1 cells to HCV, suggesting it might participate in the mechanism for self-regulation of HCV, which might be important for viral persistence. However, further studies are needed to address the precise mechanism by which core protein functions.

## Conclusions

That HCV core protein suppresses miR-122 expression may be involved in the mechanism of evading the immune response of the host.

## Methods

### Plasmids

HCV plasmid pFL-Jc1 was provided by Apath (Saint Louis, Missouri, USA). The plasmid pEGFP-core expressing GFP-tagged HCV core protein and pEGFP were constructed in our laboratory.

### Cell culture

The human hepatoma cell line Huh7.5.1 was kindly provided by Scott Forrest (Scripps Research Institute, La Jolla, CA, USA) and Jin Zhong (Institute Pasteur of Shanghai, China). Huh7.5.1 cells were maintained in Dulbecco’s Modified Eagle Medium (Gibco) supplemented with 10% fetal bovine serum (Gibco), 100 U/ml of penicillin, 100 μg/ml of streptomycin and 2 mM L-glutamine, at 37°C with 5.0% CO^2^.

### HCV Jc1 virus preparation and infection

In brief, the plasmid pFL-Jc1 was linearized by *XbaI* (New England Biolabs). Then HCV RNA was synthesized from the linearized DNA template with T7 RiboMAX™ Express Large Scale RNA Production System (Promega). Huh7.5.1 cells were regularly passaged and plated in 12-well culture plates for 20 h before transfection at 60% confluency. In vitro transcribed HCV RNA was delivered into Huh7.5.1 cells by Lipofectamine 2000 (Invitrogen), according to the manufacturer’s instructions. Transfected cells were passaged every 2–3 days before the cells became confluent. The supernatant was collected at day 3 or day 9 posttransfection and used as infectious HCV particles.

Naïve Huh7.5.1 cells plated in 12-well plate at a density of 4 × 10^4^ cells per well were incubated with culture medium containing virus (50 IU/cell) for 4 h at 37°C. Then the inoculum was discarded and fresh medium was added. Cells were passaged every 2–3 days before the cells became confluent. For each passage, half of the cell culture supernatant was discarded and fresh medium was added.

### Transfection

Huh7.5.1 cells were regularly passaged and plated in 12-well culture plates for 20 h before transfection at 60% confluency. Plasmids pEGFP-core or pEGFP were delivered into Huh7.5.1 cells by Lipofectamine 2000 (Invitrogen), according to the manufacturer’s instructions.

### Real-time PCR detection

HCV RNA in culture supernatant was quantified using diagnostic kit for quantification of hepatitis C virus RNA (Shanghai KeHua Bio-Engineering Co., Ltd.), according to the protocol. Tenfold serial dilutions of in vitro-transcribed HCV RNAs (from 10^4^-10^7^ IU/ml) served as standards, which were included in the Kit. In brief, HCV RNA in 100 μl culture supernatant was extracted using nucleic acid extraction columns included in the kit. The 12.5 μl of extracted RNA was amplified in each reaction well containing 7 μl of PCR major reaction solution, 5 μl of enzyme mixture and 0.5 μl of probe. The temperature profile comprised the RT round conducted at 50°C for 25 min and the PCR rounds denaturing at 94°C for 2 min and then at 94°C for 10 s, 55°C for 15 s and 72°C for 15 s, and completed by a 42-cycle program at 94°C for 10 s and 60°C for 45 s. Real-time PCR amplification was performed using the ABI 7500 system (Applied Biosystems).

Cellular RNA was extracted using Trizol (Invitrogen). For HCV RNA and GAPDH detection, RNA was reverse transcribed using the PrimeScript RT reagent Kit (Takara) and the cDNA production was quantified by Real-time PCR with SYBR Premix Ex Taq (Takara) according to the manufacturer’s protocol. The primers sequence for HCV were: forward primer: GCGTTAGTATGAGTGTCGTG and reverse primer: TCGCAAGCACCCTATCAG and primers sequence for GAPDH were: forward primer: GAAGGTGAAGGTCGAGTC and reverse primer: GAAGATGGTGATGGGATTTC. For miRNA detection, RT and real-time PCR were performed using miRNA analysis kits (Applied Biosystems). The stem-loop RT primers and real-time primers for miR-122 and U6 were synthesized by Ribobio (Guangzhou, China).

### Indirect immunofluorescence

Huh7.5.1 cells infected with HCV were seeded on coverslips in 12-well, then fixed in 4% paraformaldehyde for 10 min at −20°C. Fixed cells were permeabilized in 0.2% Triton-X100/PBS for 10 min at room temperature and blocked in a solution of 2% BSA/PBS for 30 min at room temperature. Then cells were incubated with anti-HCV Core antibody (Abcam) diluted 1:500, at 37°C for 2 h, followed by anti-mouse IgG conjugated to Alexa 488 (Sigma) diluted 1:500, at 37°C for 1 h. Coverslips were mounted on slides with 50% glycerol/PBS. Then, cells were examined by a Zeiss LSM 510 laser scanning confocal microscope.

### Statistical analysis

Data were given as mean ± SD obtained from three separate experiments. Statistical analysis was performed by using one-way ANOVA or by independent-sample Student’s *t*-test, using SPSS ver. 11.5 software. P-values < 0.05 were considered statistically significant.

## Competing interests

The authors declare that they have no competing interests.

## Authors’ contributions

LSJ designed and executed the experiments, analyzed the data and wrote the manuscript. XXK, YQ and XHD assisted in performing the experiments. ZHH and HJL participated in manuscript preparation and revisions. CZ and ZHH designed the experiments and provided the financial support. All authors have read and approved the final version of the manuscript.
